# *catena*-Poly[[chloridocadmium(II)]bis­{μ-1-[(2-ethyl-1*H*-imidazol-1-yl)meth­yl]-1*H*-benzotriazole}[chloridocadmium(II)]di-μ-chlorido]

**DOI:** 10.1107/S1600536811019908

**Published:** 2011-06-04

**Authors:** Xia Wang, Xian-Ju Shi, Huai-Xia Yang, Hu Feng, Pan Liu

**Affiliations:** a Pharmacy College, Henan University of Traditional Chinese Medicine, Zhengzhou 450008, People’s Republic of China; b Department of Petroleum & Chemical Engineering, Puyang Vocational and Technical College, Puyang 457000, People’s Republic of China

## Abstract

In the polymeric title complex, [CdCl_2_(C_12_H_13_N_5_)]_*n*_, the Cd^II^ atom is five-coordinated by two N atoms from two bridging 1-[(2-ethyl-1*H*-imidazol-1-yl)meth­yl]-1*H*-benzotriazole (bmei) ligands, two bridging Cl atoms and one terminal Cl atom in a distorted trigonal–bipyramidal geometry. The Cd^II^ atoms are connected alternately by the Cl atoms and bmei ligands, leading to a zigzag chain extending parallel to [011]. π–π inter­actions, with a centroid–centroid distance of 3.3016 (3) Å, help to stabilize the crystal packing.

## Related literature

For similar compounds with symmetric or asymmetric *N*-heterocyclic ligands, see: Li *et al.* (2011 ▶); Hu *et al.* (2009 ▶); Meng *et al.* (2009 ▶); Huang *et al.* (2006 ▶).
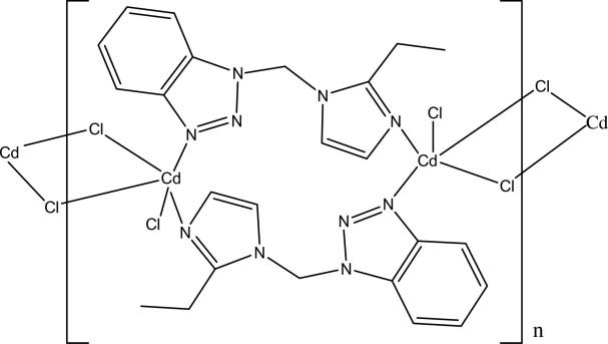


## Experimental

### 

#### Crystal data

[CdCl_2_(C_12_H_13_N_5_)]
*M* *_r_* = 410.57
Triclinic, 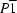

*a* = 7.6055 (6) Å
*b* = 9.7027 (11) Å
*c* = 10.3144 (10) Å
α = 74.431 (9)°
β = 81.609 (7)°
γ = 87.720 (8)°
*V* = 725.36 (12) Å^3^
*Z* = 2
Mo *K*α radiation
μ = 1.87 mm^−1^
*T* = 293 K
0.20 × 0.20 × 0.18 mm

#### Data collection

Oxford Diffraction Xcalibur Eos Gemini diffractometer
Absorption correction: multi-scan (*CrysAlis PRO*; Oxford Diffraction, 2010) ▶ *T*_min_ = 0.993, *T*_max_ = 1.000
6082 measured reflections
2963 independent reflections
2534 reflections with *I* > 2σ(*I*)
*R*_int_ = 0.027

#### Refinement

*R*[*F* ^2^ > 2σ(*F* ^2^)] = 0.035
*wR*(*F* ^2^) = 0.069
*S* = 1.02
2963 reflections
182 parameters
H-atom parameters constrained
Δρ_max_ = 0.47 e Å^−3^
Δρ_min_ = −0.50 e Å^−3^

### 

Data collection: *CrysAlis PRO* (Oxford Diffraction, 2010) ▶; cell refinement: *CrysAlis PRO*; data reduction: *CrysAlis PRO*; program(s) used to solve structure: *SHELXS97* (Sheldrick, 2008 ▶); program(s) used to refine structure: *SHELXL97* (Sheldrick, 2008 ▶); molecular graphics: *OLEX2* (Dolomanov *et al.*, 2009 ▶); software used to prepare material for publication: *publCIF* (Westrip, 2010) ▶.

## Supplementary Material

Crystal structure: contains datablock(s) global, I. DOI: 10.1107/S1600536811019908/wm2490sup1.cif

Structure factors: contains datablock(s) I. DOI: 10.1107/S1600536811019908/wm2490Isup2.hkl

Additional supplementary materials:  crystallographic information; 3D view; checkCIF report

## Figures and Tables

**Fig. 1.**
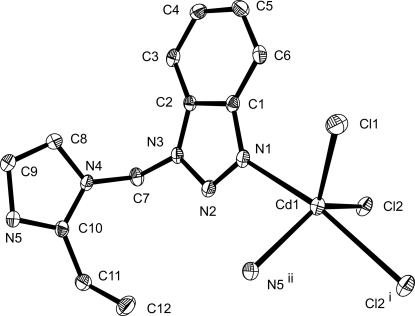
A fragment of the title complex, showing the coordination of the CdII atom with atom labelling of the non-H atoms and with 30% probability ellipsolids. [Symmetry codes: (i) -x + 1,-y + 1,-z + 1; (ii) -x + 1, -y + 2, -z.]

**Fig. 2.**
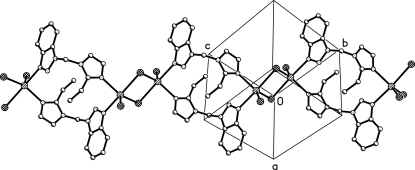
View of the zigzag chain structure of the title complex.

## supplementary crystallographic information

### Comment

In coordination and supramolecular chemistry many symmetric imidazole and benzotriazole ligands have been applied (Li *et al.*, 2011; Hu *et al.*, 2009). However, studies involving asymmetric imidazole and benzotriazole ligands are rather rare (Meng *et al.*, 2009; Huang *et al.*, 2006). We were thus engaged in the synthesis of asymmetric N-heterocyclic ligands and synthesized the compound 1-[(1*H*-benzotriazol-1-yl)methyl]-1*H*-1,3-(2-ethyl-imidazol) (bmei). In this work, we selected this compound as a ligand for generation of the new complex [Cd(C_12_H_13_N_5_)Cl_2_]*_n_*, (I), that is reported here.

In the complex (I) the Cd^II^ atom is five-coordinated by two N atoms from two bridging bmei ligands, two bridging Cl atoms and one terminal Cl atom in a distorted trigonal-bipyramidal geometry (Fig. 1). The two Cd^II^ ions are connected by a pair of bridging Cl atoms, yielding a centrosymmetric Cd_2_Cl_2_ binuclear unit with a Cd···Cd distance of 3.9657 (6) Å. The dimers are further linked by bmei ligands to give a zigzag chain extending parallel to [011] (Fig. 2). The distance between two Cd atoms bridged by the bmei ligand is 9.0727 (12) Å. In addition, the benzotriazole rings between adjacent chains are stacked in a face-to-face orientation with a centroid—centroid distance of 3.3016 (3) Å, so the crystal structure involves also π—π interactions.

### Experimental

The ligand 1-[(1*H*-benzotriazol-1-yl)methyl]-1*H*-1,3-(2-ethyl-imidazol) (0.04 mmol, 0.0096 g) in methanol (6 ml) was added dropwise to a methanol solution (5 ml) of CdCl_2_ (0.04 mmol, 0.0074 g) in methanol. The resulting solution was allowed to stand at room temperature. After one week good quality colourless crystals were obtained and dried in air.

### Refinement

H atoms were placed geometrically and refined as riding atoms with C-H = 0.93 Å and U_iso_(H) = 1.2U_eq_(C).

### Crystal data

**Table:**

[CdCl_2_(C_12_H_13_N_5_)]	*Z* = 2
*M**_r_* = 410.57	*F*(000) = 404
Triclinic, *P*1	*D*_x_ = 1.880 Mg m^−^^3^
*a* = 7.6055 (6) Å	Mo *K*α radiation, λ = 0.7107 Å
*b* = 9.7027 (11) Å	Cell parameters from 2806 reflections
*c* = 10.3144 (10) Å	θ = 3.2–26.3°
α = 74.431 (9)°	µ = 1.87 mm^−^^1^
β = 81.609 (7)°	*T* = 293 K
γ = 87.720 (8)°	Prismatic, colorless
*V* = 725.36 (12) Å^3^	0.20 × 0.20 × 0.18 mm

### Data collection

**Table:**

Oxford Diffraction Xcalibur Eos Gemini diffractometer	2963 independent reflections
Radiation source: Enhance (Mo) X-ray Source	2534 reflections with *I* > 2σ(*I*)
graphite	*R*_int_ = 0.027
Detector resolution: 16.2312 pixels mm^-1^	θ_max_ = 26.3°, θ_min_ = 3.2°
ω scans	*h* = −9→9
Absorption correction: multi-scan (*CrysAlis PRO*; Oxford Diffraction, 2010)	*k* = −12→11
*T*_min_ = 0.993, *T*_max_ = 1.000	*l* = −12→11
6082 measured reflections	

### Refinement

**Table:**

Refinement on *F*^2^	Primary atom site location: structure-invariant direct methods
Least-squares matrix: full	Secondary atom site location: difference Fourier map
*R*[*F*^2^ > 2σ(*F*^2^)] = 0.035	Hydrogen site location: inferred from neighbouring sites
*wR*(*F*^2^) = 0.069	H-atom parameters constrained
*S* = 1.02	*w* = 1/[σ^2^(*F*_o_^2^) + (0.0217*P*)^2^] where *P* = (*F*_o_^2^ + 2*F*_c_^2^)/3
2963 reflections	(Δ/σ)_max_ = 0.001
182 parameters	Δρ_max_ = 0.47 e Å^−^^3^
0 restraints	Δρ_min_ = −0.50 e Å^−^^3^

### Special details

**Table:**

Geometry. All e.s.d.'s (except the e.s.d. in the dihedral angle between two l.s. planes) are estimated using the full covariance matrix. The cell e.s.d.'s are taken into account individually in the estimation of e.s.d.'s in distances, angles and torsion angles; correlations between e.s.d.'s in cell parameters are only used when they are defined by crystal symmetry. An approximate (isotropic) treatment of cell e.s.d.'s is used for estimating e.s.d.'s involving l.s. planes.
Refinement. Refinement of *F*^2^ against ALL reflections. The weighted *R*-factor *wR* and goodness of fit *S* are based on *F*^2^, conventional *R*-factors *R* are based on *F*, with *F* set to zero for negative *F*^2^. The threshold expression of *F*^2^ > σ(*F*^2^) is used only for calculating *R*-factors(gt) *etc*. and is not relevant to the choice of reflections for refinement. *R*-factors based on *F*^2^ are statistically about twice as large as those based on *F*, and *R*- factors based on ALL data will be even larger.

### Fractional atomic coordinates and isotropic or equivalent isotropic displacement parameters (Å^2^)

**Table:**

	*x*	*y*	*z*	*U*_iso_*/*U*_eq_	
Cd1	0.37119 (4)	0.60250 (3)	0.34830 (3)	0.02594 (10)	
Cl1	0.10727 (13)	0.49171 (10)	0.30891 (11)	0.0366 (3)	
Cl2	0.41293 (13)	0.62093 (9)	0.58398 (10)	0.0321 (2)	
N1	0.2477 (4)	0.8391 (3)	0.3168 (3)	0.0266 (7)	
N2	0.3564 (4)	0.9476 (3)	0.2763 (3)	0.0270 (7)	
N3	0.2578 (4)	1.0677 (3)	0.2711 (3)	0.0240 (7)	
N4	0.3531 (4)	1.2614 (3)	0.0745 (3)	0.0268 (7)	
N5	0.4535 (4)	1.3280 (3)	−0.1440 (3)	0.0302 (8)	
C1	0.0746 (5)	0.8865 (4)	0.3398 (4)	0.0241 (8)	
C2	0.0805 (5)	1.0354 (4)	0.3106 (4)	0.0223 (8)	
C3	−0.0701 (5)	1.1177 (4)	0.3322 (4)	0.0267 (8)	
H3	−0.0656	1.2167	0.3151	0.032*	
C4	−0.2255 (5)	1.0418 (4)	0.3806 (4)	0.0303 (9)	
H4	−0.3299	1.0915	0.3969	0.036*	
C5	−0.2333 (5)	0.8923 (4)	0.4064 (4)	0.0322 (9)	
H5	−0.3426	0.8466	0.4374	0.039*	
C6	−0.0851 (5)	0.8122 (4)	0.3874 (4)	0.0281 (9)	
H6	−0.0904	0.7132	0.4052	0.034*	
C7	0.3445 (5)	1.2069 (4)	0.2205 (4)	0.0294 (9)	
H7B	0.2797	1.2742	0.2643	0.035*	
H7A	0.4640	1.1987	0.2442	0.035*	
C8	0.2094 (5)	1.3085 (4)	0.0082 (4)	0.0345 (10)	
H8	0.0919	1.3119	0.0479	0.041*	
C9	0.2719 (5)	1.3492 (4)	−0.1261 (4)	0.0382 (10)	
H9	0.2035	1.3856	−0.1954	0.046*	
C10	0.4998 (5)	1.2744 (4)	−0.0210 (4)	0.0266 (8)	
C11	0.6844 (5)	1.2391 (4)	0.0090 (4)	0.0365 (10)	
H11B	0.7009	1.2715	0.0876	0.044*	
H11A	0.7665	1.2922	−0.0674	0.044*	
C12	0.7329 (6)	1.0793 (4)	0.0370 (4)	0.0457 (11)	
H12A	0.7140	1.0451	−0.0389	0.069*	
H12B	0.6593	1.0264	0.1173	0.069*	
H12C	0.8555	1.0668	0.0502	0.069*	

### Atomic displacement parameters (Å^2^)

**Table:**

	*U*^11^	*U*^22^	*U*^33^	*U*^12^	*U*^13^	*U*^23^
Cd1	0.02975 (16)	0.02168 (16)	0.02519 (18)	0.00200 (11)	−0.00631 (12)	−0.00314 (12)
Cl1	0.0331 (5)	0.0287 (5)	0.0511 (7)	−0.0001 (4)	−0.0110 (5)	−0.0133 (5)
Cl2	0.0412 (6)	0.0275 (5)	0.0290 (6)	0.0108 (4)	−0.0100 (4)	−0.0087 (4)
N1	0.0333 (18)	0.0213 (16)	0.0227 (19)	0.0029 (14)	−0.0050 (14)	−0.0014 (13)
N2	0.0319 (18)	0.0236 (16)	0.0238 (19)	0.0048 (14)	−0.0044 (14)	−0.0038 (13)
N3	0.0314 (18)	0.0182 (15)	0.0197 (18)	0.0015 (13)	−0.0017 (14)	−0.0017 (13)
N4	0.0287 (17)	0.0235 (16)	0.0251 (19)	0.0018 (13)	−0.0074 (14)	0.0005 (13)
N5	0.0283 (18)	0.0332 (18)	0.0240 (19)	0.0025 (14)	−0.0036 (14)	0.0009 (14)
C1	0.031 (2)	0.0235 (19)	0.017 (2)	0.0042 (16)	−0.0018 (16)	−0.0045 (15)
C2	0.0267 (19)	0.0219 (19)	0.017 (2)	0.0004 (15)	−0.0028 (15)	−0.0036 (15)
C3	0.037 (2)	0.0228 (19)	0.021 (2)	0.0059 (17)	−0.0074 (17)	−0.0064 (16)
C4	0.027 (2)	0.036 (2)	0.028 (2)	0.0062 (17)	−0.0050 (17)	−0.0095 (18)
C5	0.029 (2)	0.036 (2)	0.029 (2)	−0.0055 (18)	−0.0010 (18)	−0.0051 (18)
C6	0.035 (2)	0.0225 (19)	0.025 (2)	−0.0036 (17)	−0.0068 (17)	−0.0011 (16)
C7	0.040 (2)	0.025 (2)	0.023 (2)	−0.0029 (17)	−0.0034 (18)	−0.0063 (17)
C8	0.027 (2)	0.036 (2)	0.035 (3)	0.0032 (18)	−0.0033 (18)	−0.0013 (19)
C9	0.030 (2)	0.045 (2)	0.032 (3)	0.0039 (19)	−0.0086 (19)	0.004 (2)
C10	0.030 (2)	0.0191 (18)	0.028 (2)	0.0003 (16)	−0.0031 (17)	−0.0019 (16)
C11	0.029 (2)	0.047 (3)	0.029 (2)	−0.0005 (19)	−0.0057 (18)	−0.002 (2)
C12	0.046 (3)	0.056 (3)	0.034 (3)	0.021 (2)	−0.012 (2)	−0.009 (2)

### Geometric parameters (Å, °)

**Table:**

Cd1—Cl1	2.4482 (10)	C3—H3	0.9300
Cd1—Cl2^i^	2.6687 (10)	C3—C4	1.374 (5)
Cd1—Cl2	2.5505 (10)	C4—H4	0.9300
Cd1—N1	2.403 (3)	C4—C5	1.405 (5)
Cd1—N5^ii^	2.272 (3)	C5—H5	0.9300
Cl2—Cd1^i^	2.6686 (10)	C5—C6	1.363 (5)
N1—N2	1.303 (4)	C6—H6	0.9300
N1—C1	1.386 (5)	C7—H7B	0.9700
N2—N3	1.353 (4)	C7—H7A	0.9700
N3—C2	1.374 (4)	C8—H8	0.9300
N3—C7	1.457 (4)	C8—C9	1.353 (5)
N4—C7	1.449 (5)	C9—H9	0.9300
N4—C8	1.371 (5)	C10—C11	1.488 (5)
N4—C10	1.362 (5)	C11—H11B	0.9700
N5—Cd1^ii^	2.272 (3)	C11—H11A	0.9700
N5—C9	1.381 (5)	C11—C12	1.539 (5)
N5—C10	1.329 (5)	C12—H12A	0.9600
C1—C2	1.395 (5)	C12—H12B	0.9600
C1—C6	1.394 (5)	C12—H12C	0.9600
C2—C3	1.396 (5)		
			
Cd1—Cl2—Cd1^i^	98.87 (3)	C3—C4—H4	118.6
Cl1—Cd1—Cl2^i^	102.49 (3)	C3—C4—C5	122.8 (4)
Cl1—Cd1—Cl2	121.87 (4)	C4—C3—C2	115.1 (3)
Cl2—Cd1—Cl2^i^	81.13 (3)	C4—C3—H3	122.5
N1—Cd1—Cl1	95.86 (8)	C4—C5—H5	119.1
N1—Cd1—Cl2	85.51 (8)	C5—C4—H4	118.6
N1—Cd1—Cl2^i^	161.13 (8)	C5—C6—C1	116.5 (3)
N1—N2—N3	107.5 (3)	C5—C6—H6	121.7
N1—C1—C2	107.3 (3)	C6—C1—C2	121.3 (3)
N1—C1—C6	131.4 (3)	C6—C5—C4	121.8 (4)
N2—N1—Cd1	118.2 (2)	C6—C5—H5	119.1
N2—N1—C1	110.0 (3)	H7B—C7—H7A	107.9
N2—N3—C2	111.1 (3)	C8—N4—C7	124.7 (3)
N2—N3—C7	119.4 (3)	C8—C9—N5	109.1 (4)
N3—C2—C1	104.2 (3)	C8—C9—H9	125.4
N3—C2—C3	133.2 (3)	C9—N5—Cd1^ii^	123.7 (3)
N3—C7—H7B	109.2	C9—C8—N4	106.6 (4)
N3—C7—H7A	109.2	C9—C8—H8	126.7
N4—C7—N3	112.0 (3)	C10—N4—C7	127.5 (3)
N4—C7—H7B	109.2	C10—N4—C8	107.9 (3)
N4—C7—H7A	109.2	C10—N5—Cd1^ii^	129.2 (3)
N4—C8—H8	126.7	C10—N5—C9	106.8 (3)
N4—C10—C11	124.9 (4)	C10—C11—H11B	108.5
N5^ii^—Cd1—Cl1	106.55 (8)	C10—C11—H11A	108.5
N5^ii^—Cd1—Cl2^i^	88.42 (8)	C10—C11—C12	115.0 (3)
N5^ii^—Cd1—Cl2	131.58 (8)	C11—C12—H12A	109.5
N5^ii^—Cd1—N1	90.57 (11)	C11—C12—H12B	109.5
N5—C9—H9	125.4	C11—C12—H12C	109.5
N5—C10—N4	109.5 (3)	H11B—C11—H11A	107.5
N5—C10—C11	125.5 (4)	C12—C11—H11B	108.5
C1—N1—Cd1	131.7 (2)	C12—C11—H11A	108.5
C1—C2—C3	122.5 (3)	H12A—C12—H12B	109.5
C1—C6—H6	121.7	H12A—C12—H12C	109.5
C2—N3—C7	129.5 (3)	H12B—C12—H12C	109.5
C2—C3—H3	122.5		
			
Cd1—N1—N2—N3	−177.7 (2)	N4—C10—C11—C12	81.6 (5)
Cd1—N1—C1—C2	176.9 (2)	N5^ii^—Cd1—Cl2—Cd1^i^	−79.87 (11)
Cd1—N1—C1—C6	−0.9 (6)	N5^ii^—Cd1—N1—N2	−47.0 (3)
Cd1^ii^—N5—C9—C8	−174.2 (2)	N5^ii^—Cd1—N1—C1	136.3 (3)
Cd1^ii^—N5—C10—N4	173.7 (2)	N5—C10—C11—C12	−100.9 (4)
Cd1^ii^—N5—C10—C11	−4.0 (5)	C1—N1—N2—N3	−0.3 (4)
Cl1—Cd1—Cl2—Cd1^i^	99.25 (4)	C1—C2—C3—C4	−1.8 (5)
Cl1—Cd1—N1—N2	−153.7 (2)	C2—N3—C7—N4	−87.5 (4)
Cl1—Cd1—N1—C1	29.6 (3)	C2—C1—C6—C5	−1.4 (5)
Cl2^i^—Cd1—Cl2—Cd1^i^	0.000 (2)	C2—C3—C4—C5	−0.1 (6)
Cl2^i^—Cd1—N1—N2	39.8 (4)	C3—C4—C5—C6	1.3 (6)
Cl2—Cd1—N1—N2	84.7 (2)	C4—C5—C6—C1	−0.5 (6)
Cl2—Cd1—N1—C1	−92.0 (3)	C6—C1—C2—N3	178.3 (3)
Cl2^i^—Cd1—N1—C1	−136.9 (3)	C6—C1—C2—C3	2.7 (6)
N1—Cd1—Cl2—Cd1^i^	−166.64 (8)	C7—N3—C2—C1	175.8 (3)
N1—N2—N3—C2	0.5 (4)	C7—N3—C2—C3	−9.3 (6)
N1—N2—N3—C7	−176.2 (3)	C7—N4—C8—C9	−179.5 (3)
N1—C1—C2—N3	0.3 (4)	C7—N4—C10—N5	179.6 (3)
N1—C1—C2—C3	−175.3 (3)	C7—N4—C10—C11	−2.6 (6)
N1—C1—C6—C5	176.1 (4)	C8—N4—C7—N3	70.3 (4)
N2—N1—C1—C2	0.0 (4)	C8—N4—C10—N5	0.1 (4)
N2—N1—C1—C6	−177.7 (4)	C8—N4—C10—C11	177.9 (3)
N2—N3—C2—C1	−0.5 (4)	C9—N5—C10—N4	−0.2 (4)
N2—N3—C2—C3	174.4 (4)	C9—N5—C10—C11	−178.0 (3)
N2—N3—C7—N4	88.6 (4)	C10—N4—C7—N3	−109.1 (4)
N3—C2—C3—C4	−176.0 (4)	C10—N4—C8—C9	0.0 (4)
N4—C8—C9—N5	−0.1 (4)	C10—N5—C9—C8	0.2 (4)

Symmetry codes: (i) −*x*+1, −*y*+1, −*z*+1; (ii) −*x*+1, −*y*+2, −*z*.

## References

[bb1] Dolomanov, O. V., Bourhis, L. J., Gildea, R. J., Howard, J. A. K. & Puschmann, H. (2009). *J. Appl. Cryst.* **42**, 339–341.

[bb2] Hu, M.-C., Wang, Y., Zhai, Q.-G., Li, S.-N., Jiang, Y.-C. & Zhang, Y. (2009). *Inorg. Chem.* **48**, 1449–1468. 10.1021/ic801574k 19154122

[bb3] Huang, M.-H., Liu, P., Wang, J., Chen, Y. & Liu, Q.-Y. (2006). *Inorg. Chem. Commun.* **9**, 952–954.

[bb4] Li, B.-Y., Yang, F., Li, G.-H., Liu, D., Zhou, Q., Shi, Z. & Feng, S.-H. (2011). *Cryst. Growth Des.* **11**, 1475–1485.

[bb5] Meng, X.-R., Jin, S.-Z., Hou, H.-W., Du, C.-X. & Ng, S. W. (2009). *Inorg. Chim. Acta*, **362**, 1519–1527.

[bb6] Oxford Diffraction (2010). *CrysAlis PRO* Oxford Diffraction Ltd, Yarnton, England.

[bb7] Sheldrick, G. M. (2008). *Acta Cryst.* A**64**, 112–122. 10.1107/S0108767307043930 18156677

[bb8] Westrip, S. P. (2010). *J. Appl. Cryst.* **43**, 920–925.

